# LncRNA HOXA-AS3 promotes cell proliferation and invasion via targeting miR-218-5p/FOXP1 axis in osteosarcoma

**DOI:** 10.1038/s41598-024-67596-4

**Published:** 2024-07-17

**Authors:** Rong Li, Pingbo Chen, Yubo Zhou, Yi Lang, Changhui Zhou, Jingqin Ren, Adilijiang Maimaitiyimin, Zhen Chen, Chengqing Liu, Abasi mainike, Lu Ding

**Affiliations:** 1 College of Public Health, State Key Laboratory of Special Environment and Health Research in Xinjiang, Xinjiang Medical University, Urumqi, Xinjiang China; 2 Traditional Chinese Medicine Hospital Affiliated to Xinjiang Medical University, Urumqi, 830017 Xinjiang China; 3 Xinjiang Medical University Affiliated Fifth Hospital, Urumqi, Xinjiang China; 4 Postdoctoral Research Center on Public Health and Preventive Medicine, Xinjiang Medical University, Urumqi, Xinjiang China

**Keywords:** Osteosarcoma, miR-218-5p, HOXA-AS3, FOXP1, Invasion, Bone cancer, Bone cancer, Gene silencing

## Abstract

Osteosarcoma is an aggressive form of bone cancer and affects the health in children and adolescents. Although conventional treatment improves the osteosarcoma survival, some patients have metastasis and drug resistance, leading to a worse prognosis. Therefore, it is necessary to explore the molecular mechanism of osteosarcoma occurrence and progression, which could discover the novel treatment for osteosarcoma. Long noncoding RNAs (lncRNAs) have been reported to regulate osteosarcoma occurrence and malignant progression. LncRNA HOXA-AS3 facilitates the tumorigenesis and progression in a variety of human cancers. However, the underlying mechanism of lncRNA HOXA-AS3-induced oncogenesis is poorly determined in osteosarcoma. To address this point, we utilized several cellular biological strategies and molecular approaches to explore the biological functions and mechanisms of lncRNA HOXA-AS3 in osteosarcoma cells. We found that lncRNA HOXA-AS3 facilitates cell proliferation and invasion via targeting miR-218-5p/FOXP1 axis in osteosarcoma. In conclusion, lncRNA HOXA-AS3 could be a promising target for osteosarcoma treatment.

## Introduction

Osteosarcoma is an aggressive form of bone cancer, which often affects the long bones, including the legs and arms^1,2^. Osteosarcoma is commonly diagnosed in children and adolescents. Osteosarcoma patients have common symptoms, such as pain, swelling, limited joint movement and bone fractures^3,4^. The treatments of osteosarcoma include surgery, chemotherapy, radiation therapy and immunotherapy^5–7^. Although early detection and conventional treatment have improved the survival rate of osteosarcoma, some patients have a worse prognosis due to drug resistance and distant metastasis^8^. Therefore, it is pivotal to explore the molecular mechanisms of osteosarcoma occurrence and progression and uncover the novel treatment^9–11^.

It has been documented that noncoding RNAs (ncRNAs) regulate tumor development and progression^12,13^. Noncoding RNAs are divided into several groups: long ncRNAs (more than 200 nucleotides) and small ncRNAs (less than 200 nucleotides)^14–16^. Evidence has uncovered that ncRNAs regulate cell growth, invasion, metastasis, apoptosis, autophagy, drug resistance, and immunotherapy in human cancer^17,18^. LncRNA HOXA-AS3 (HOXA cluster antisense RNA 3) has been reported to regulate specific targets and participate in disease development^19,20^. For instance, lncRNA HOXA-AS3 regulated lineage commitment of mesenchymal stem cells via interaction with EZH2 (Enhancer of Zeste 2)^21^. LncRNA HOXA-AS3 was found to regulate endothelium inflammation via integrating NF-kappaB signaling pathway and regulating the expression of IκBα and p65 acetylation^22^. LncRNA HOXA-AS3 was identified in endometriosis development via construction of ceRNA network^23^. One study showed that lncRNA HOXA-AS3 regulated the miR-675-3p and PDE5A (phosphodiesterase 5A) expression and accelerated the progression of pulmonary arterial hypertension^24^. Another study showed that downregulation of lncRNA HOXA-AS3 increased the expression of miR-455-5p and reduced the expression of p27, which reduced the atherosclerosis progression^25^.

Wu et al. reported that overexpression of lncRNA HOXA-AS3 facilitated tumor malignant progression via regulation of cell apoptosis and cell cycle progression as well as cell migration in glioma. The glioma patients with higher expression of lncRNA HOXA-AS3 displayed a poor prognosis^26^. In lung adenocarcinoma, lncRNA HOXA-AS3 enhanced the stability of HOXA6 mRNA via formation of an RNA duplex. LncRNA HOXA-AS3 accelerated invasion, migration and progression via binding with NF110. Moreover, NF110 governed the subcellular distribution of HOXA-AS3 in A549 cells^27^. In hepatocellular carcinoma (HCC), lncRNA HOXA-AS3 was reported to promote cell metastasis, EMT and proliferation via enhancing the activation of the mitogen-activated protein kinase (MAPK) and extracellular regulated protein kinase (ERK) through sponging miR-29c^28^. Similarly, lncRNA HOXA-AS3 was demonstrated to increase cell migration and proliferation via regulating miR-455-5p and PD-L1 axis in HCC cells^29^. LncRNA HOXA-AS3 was reported to involve in osteogenesis ossification from mesenchymal stem cells^30^. Xiao et al. reported that lncRNA HOXA-AS3 targeted the miR-1286 and TEAD1 axis, leading to promotion of osteosarcoma progression^31^. However, the molecular mechanism of lncRNA HOXA-AS3-mediated oncogenesis is poorly explored in human osteosarcoma. In this present study, we utilized several cellular biological strategies to explore the biological function of lncRNA HOXA-AS3 in osteosarcoma cells. We also utilized several molecular biological strategies to determine the underlying mechanism of lncRNA HOXA-AS3-mediated oncogenesis in osteosarcoma.

## Results

### Inhibition of lncRNA HOXA-AS3 reduces colony formation

**Figure 1:**
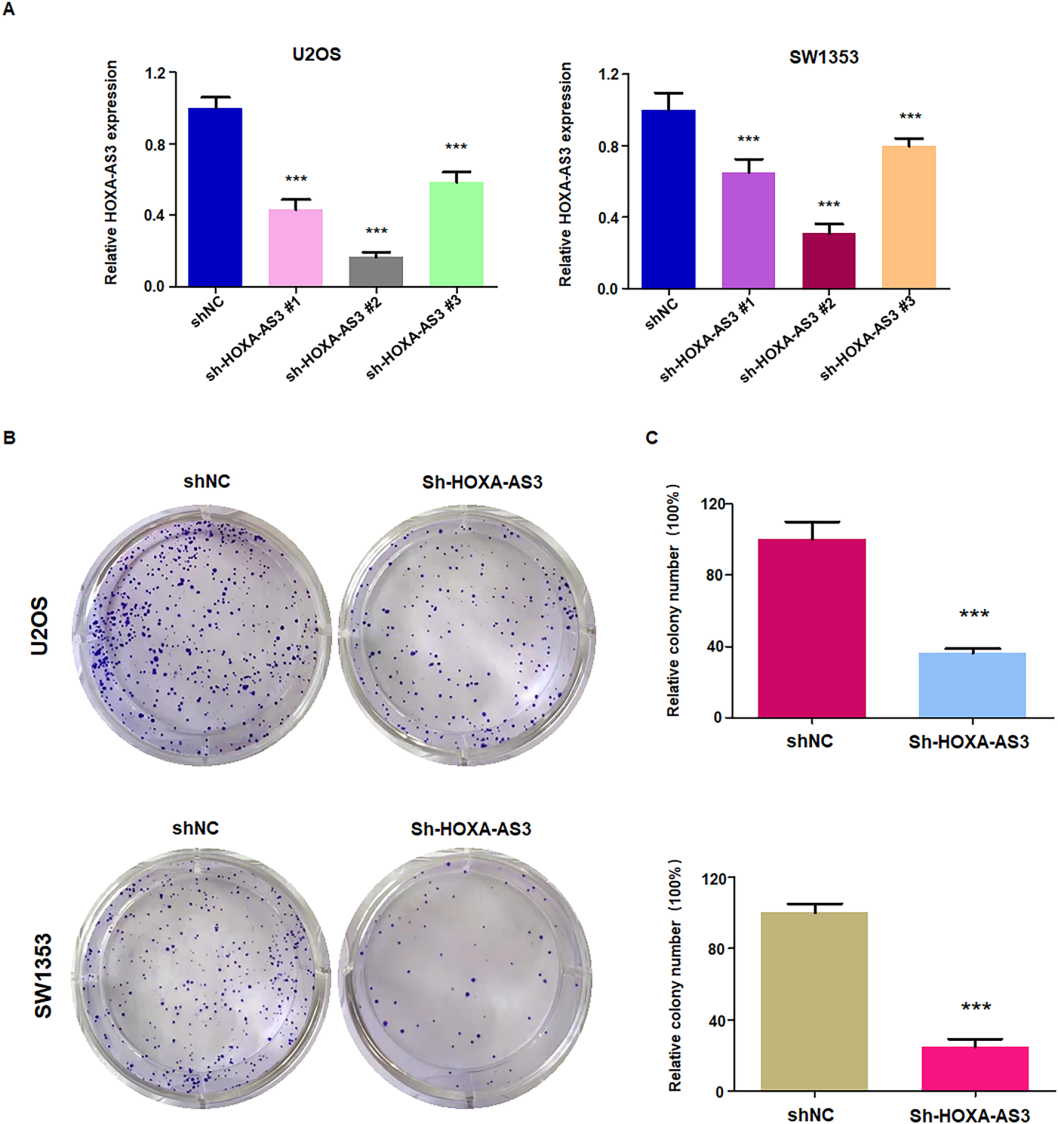
Downregulation of lncRNA HOXA-AS3 reduces colony formation of osteosarcoma cells. (**A**) RT-PCR data showed that sh-HOXA-AS3 transfection reduced the expression of HOXA-AS3 in U2OS and SW1353 cells. (**B**) Left panel: Colony formation assays showed that HOXA-AS3 transfection reduced colony formation in U2OS and SW1353 cells. Right panel: quantitative data are shown for colony formation. ***p < 0.001 versus control group.

To explore the function of lncRNA HOXA-AS3 on proliferation of U2OS and SW1353 cells, we used shRNA approach to downregulate the expression of lncRNA HOXA-AS3. The RT-PCR results showed that lncRNA HOXA-AS3 expression was significantly downregulated in U2OS and SW1353 cells (Fig. 1A). Among three shRNAs for depletion of HOXA-AS3, sh-HOXA-AS3 #2 exhibited the powerful for downregulation of lncRNA HOXA-AS3. Hence, we used sh-HOXA-AS3 #2 in the flowing experiments to knockdown the expression of lncRNA HOXA-AS3. To determine the function of lncRNA HOXA-AS3 on osteosarcoma cells, colony formation experiments were performed in U2OS and SW1353 cells after sh-HOXA-AS3 transfection. We found that the number of colony formation was decreased in both U2OS and SW1353 cell lines after HOXA-AS3 expression was reduced (Fig. 1B). This study suggested that inhibition of lncRNA HOXA-AS3 reduced colony formation of osteosarcoma cells.

### Inhibition of lncRNA HOXA-AS3 reduces migration and invasion

**Figure 2:**
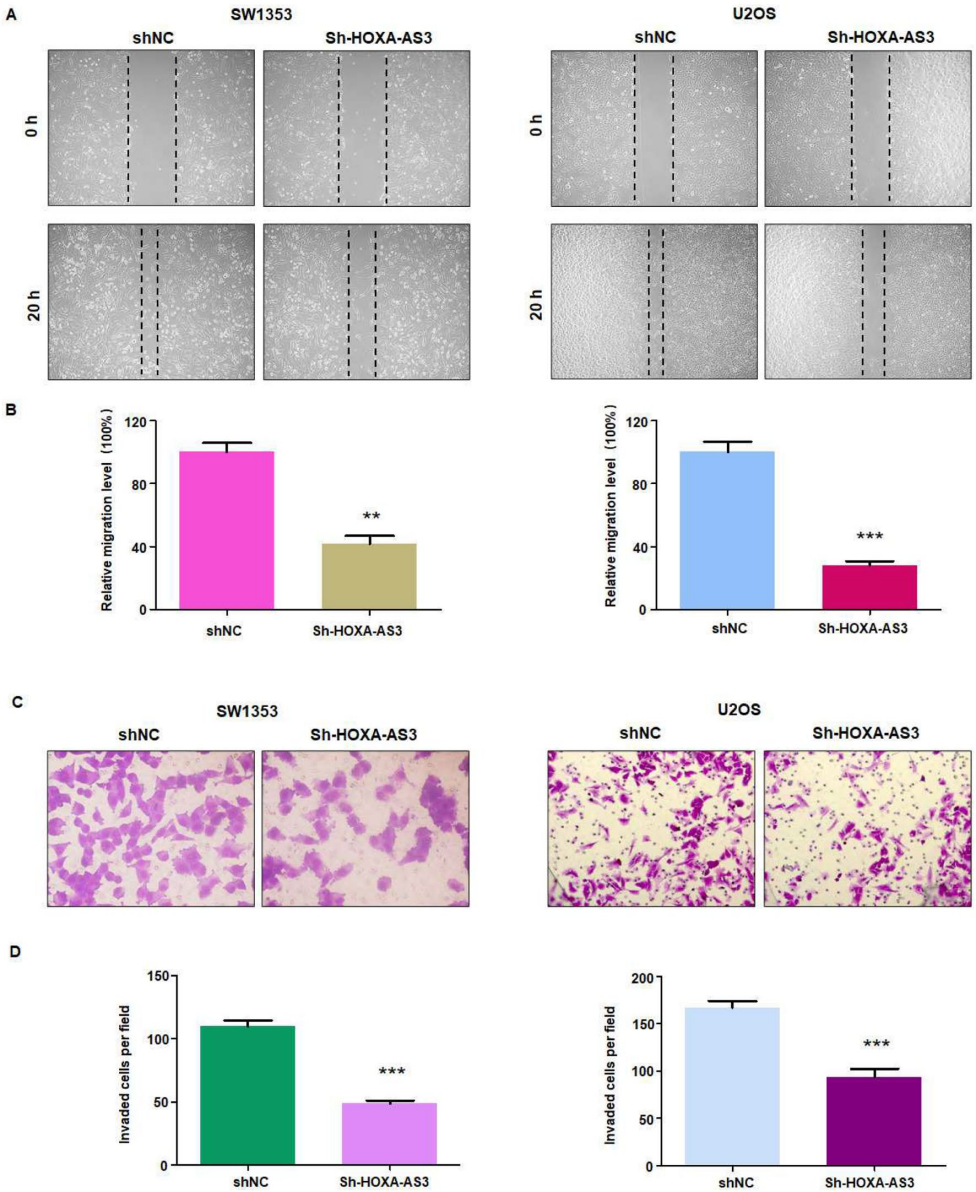
Downregulation of lncRNA HOXA-AS3 reduces migration and invasion of osteosarcoma cells. (**A**) Wound healing assays showed that sh-HOXA-AS3 transfection inhibited migratory ability of SW1353 and U2OS cells. (**B**) Quantitative data are shown for wound healing assay. (**C**) Transwell invasion assays showed that sh-HOXA-AS3 transfection reduced invasion of SW1353 and U2OS cells. (**D**) Quantitative data are shown for invasion ability of osteosarcoma cells. **p < 0.01; ***p < 0.001 versus control group.

It is known that lncRNA HOXA-AS3 downregulation inhibited the cell invasion and migration in cervical cancer cells via targeting miR-29a-3p^32^. LncRNA HOXA-AS3 downregulation reduced migration and invasion via targeting miR-29a-3p in gastric cancer^33^. In addition, lncRNA HOXA-AS3 promoted migration and invasion of A549 lung cancer cells^27^. Therefore, we used wound healing assay and Transwell invasion assay to measure the cell migratory and invasive abilities in osteosarcoma cancer after lncRNA HOXA-AS3 downregulation. As shown in Fig. 2A and B, sh-HOXA-AS3 transfection reduced the wound healing, suggesting that lncRNA HOXA-AS3 downregulation inhibited the cell migratory ability in SW1353 and U2OS cells. Moreover, Transwell assay data demonstrated that sh-HOXA-AS3 transfection reduced the number of invaded cells in SW1353 and U2OS cells (Fig. 2C,D). Taken together, inhibition of lncRNA HOXA-AS3 attenuated migration and invasion of osteosarcoma cells.

### LncRNA HOXA-AS3 interacts with miR-218-5p

**Figure 3:**
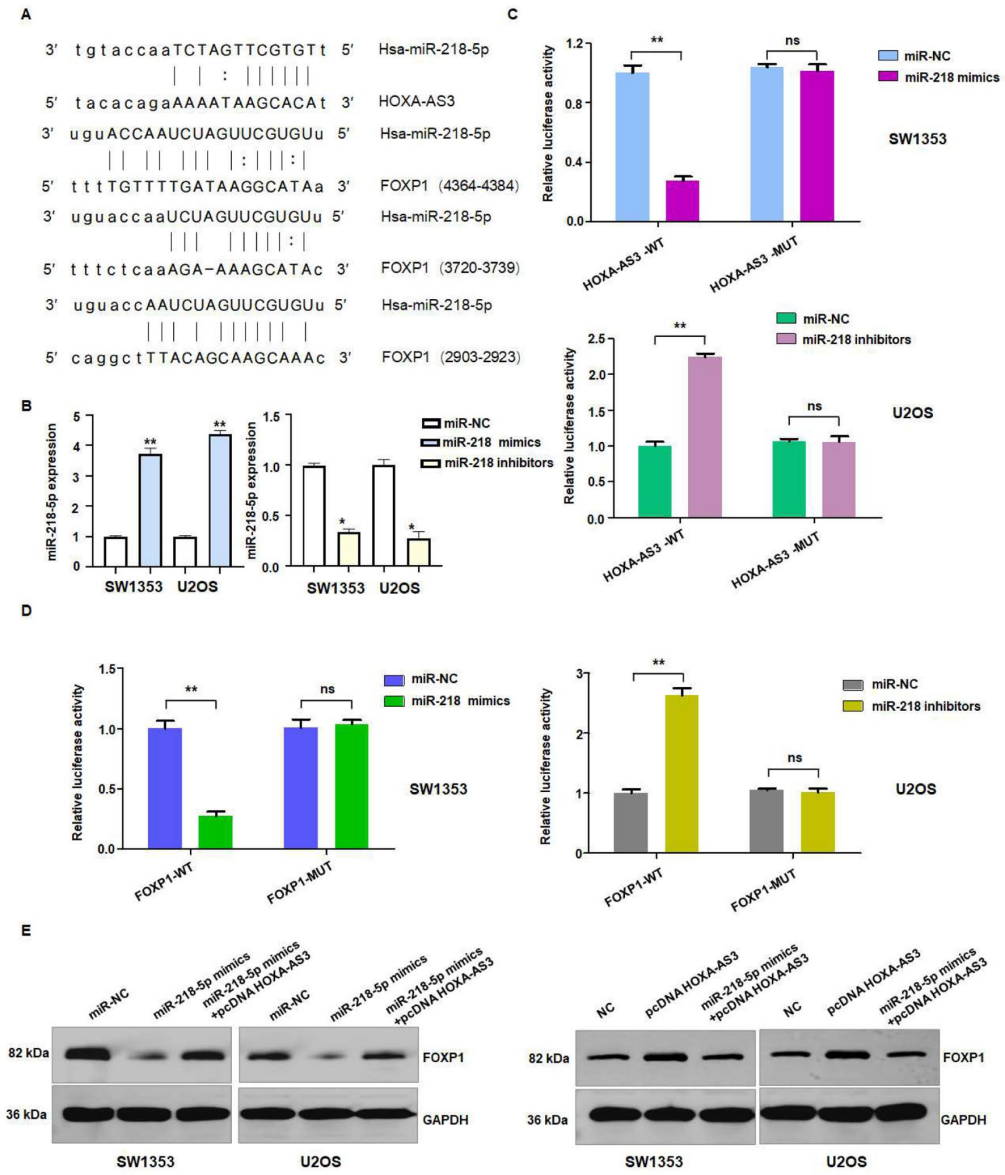
LncRNA HOXA-AS3 interacts with miR-218-5p in osteosarcoma cells. (**A**) Potential binding sites between miR-218-5p and lncRNA HOXA-AS3 and FOXP1. (**B**) RT-PCR data showed that miR-218-5p inhibitors decreased the expression of miR-218-5p. In addition, miR-218-5p mimic transfection increased the miR-218-5p expression levels. (**C**) Dual luciferase reporter assays showed that miR-218-5p interacted with lncRNA HOXA-AS3. miR-218-5p regulated luciferase activity in HOXA-AS3 wild-type group. (**D**) Dual luciferase reporter assays showed that miR-218-5p interacted with and regulated FOXP1. **p < 0.01 versus control group. (**E**) Western blotting analysis data showed that lncRNA HOXA-AS3 and miR-218-5p regulate the expression of FOXP1 in osteosarcoma cells. WT: wild type; MUT: mutant.

It has been documented that lncRNAs perform their functions in part via sponging miRNAs to reduce the miRNA target’s expression. Hence, we determined the miRNAs that can interact with lncRNA HOXA-AS3 in osteosarcoma. Several studies have shown the critical role of miR-218-5p in osteosarcoma development^34–36^. According to the TargetScan database, we found the interacting sites between lncRNA HOXA-AS3 and miR-218-5p (Fig. 3A). Our result showed that miR-218-5p inhibitors treatment suppressed the expression of miR-218-5p, while miR-218-5p mimic increased the expression of miR-218-5p (Fig. 3B). To validate the binding sites between miR-218-5p and HOXA-AS3, we did the luciferase reporter gene assay. We found that miR-218-5p mimic decreased the luciferase activity in the lncRNA HOXA-AS3 wild-type group, while HOXA-AS3 mutant group did not exhibit the change of luciferase activity (Fig. 3C). Furthermore, miR-218-5p inhibitor treatment elevated the luciferase activity in the HOXA-AS3 wild-type group, whereas miR-218-5p inhibitor did not change the luciferase activity in the HOXA-AS3 mutant group (Fig. 3C). Hence, lncRNA HOXA-AS3 could interact with miR-218-5p in osteosarcoma cells.

### FOXP1 interacts with miR-218-5p

FOXP1 has been reported to drive osteosarcoma development via inhibition of p21, retinoblastoma protein (RB) transcription and inactivation of p53^37^. To explore the potential target of miR-218-5p, we used TargetScan database and found that FOXP1 could be a potential target of miR-218-5p because there are several interacting sites between FOXP1 and miR-218-5p (Fig. 3A). The luciferase assay data revealed that miR-218-5p mimics decreased the luciferase activity in the FOXP1 wild-type group, while FOXP1 mutant group did not exhibit the change of luciferase activity (Fig. 3D). Furthermore, miR-218-5p inhibitor treatment elevated the luciferase activity in the FOXP1 wild-type group, whereas miR-218-5p inhibitor did not change the luciferase activity in the FOXP1 mutant group (Fig. 3D). Our western blotting data showed that miR-218-5p mimic transfection inhibited the expression of FOXP1 in SW1353 cells, while HOXA-AS3 upregulation increased the expression of FOXP1 in U2OS cells (Fig. 3E). Overexpression of HOXA-AS3 abrogated the miR-218-5p mimic-mediated inhibition of FOXP1 expression in SW1353 cells. Similarly, miR-218-5p mimics abolished the pcDNA HOXA-AS3-mediated inhibition of FOXP1 in U2OS cells (Fig. 3E). Hence, lncRNA HOXA-AS3 could regulate FOXP1 in osteosarcoma cells.

### Downregulation of lncRNA HOXA-AS3 inhibits colony formation via miR-218-5p

**Figure 4:**
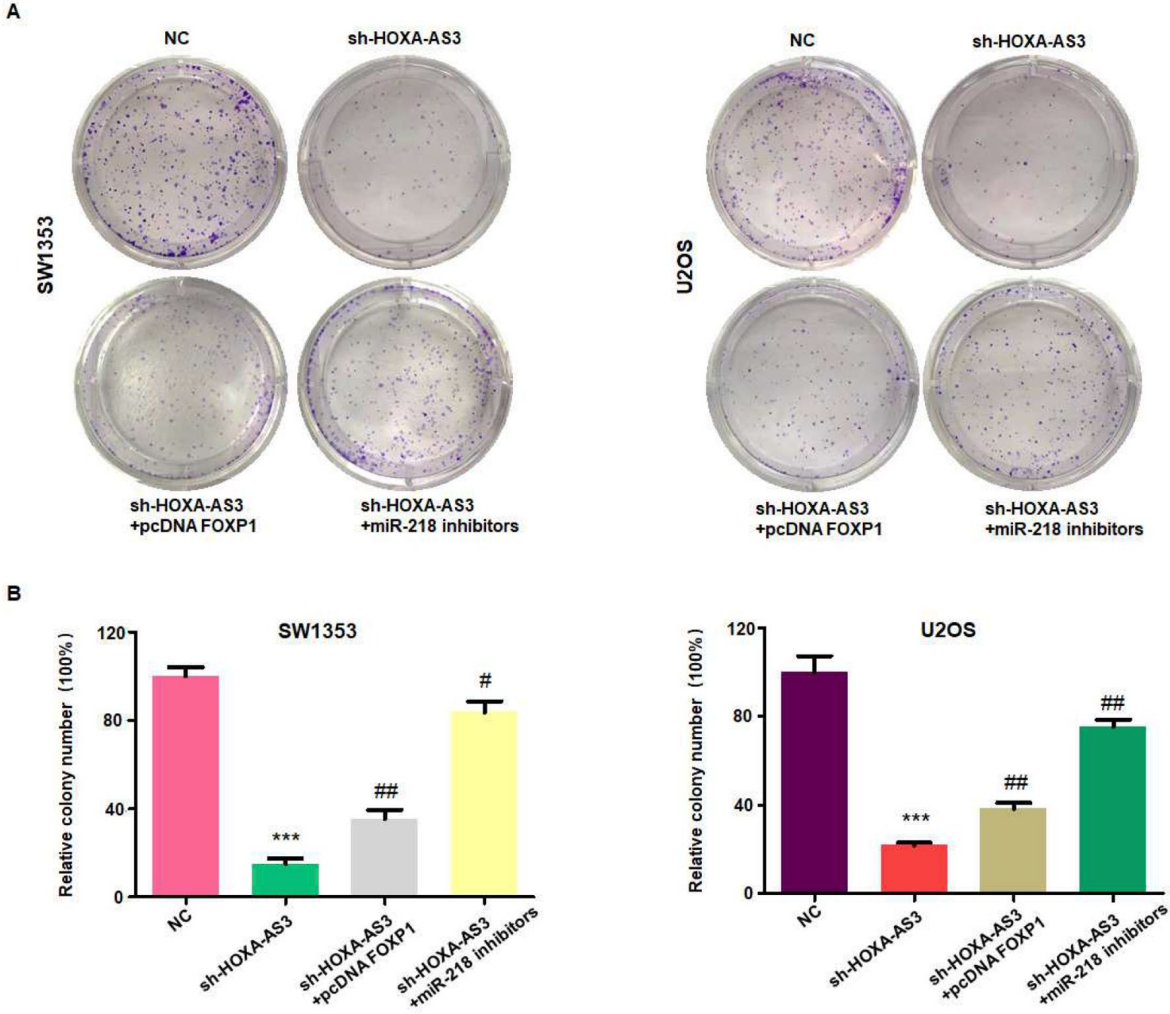
Downregulation of lncRNA HOXA-AS3 reduces colony formation via miR-218-5p and FOXP1. (**A**) Colony formation assays were performed to measure the colony formation in osteosarcoma cells co-transfected with sh-HOXA-AS3 and pcDNA FOXP1, or sh-HOXA-AS3 and miR-218 inhibitors. (**B**) Quantitative data are shown for colony formation. ***p < 0.001 versus control group. ^#^*p* < 0.05; ^##^*p* < 0.01 versus sh-HOXA-AS3 alone.

To test whether lncRNA HOXA-AS3 regulates colony formation of osteosarcoma cells via miR-218-5p, sh-HOXA-AS3 and miR-218-5p inhibitor were co-transfected into osteosarcoma cells. We found that sh-HOXA-AS3 transfection inhibited colony formation in SW1353 and U2OS cells (Fig. 4A,B). Inhibition of miR-218-5p by its inhibitor abrogated sh-HOXA-AS3-mediated inhibition of colony formation in osteosarcoma cells (Fig. 4A,B). Likewise, overexpression of FOXP1 by pcDNA FOXP1 transfection abrogated sh-HOXA-AS3-induced inhibition of colony formation in osteosarcoma cells (Fig. 4A,B). Taken together, downregulation of lncRNA HOXA-AS3 reduced colony formation via miR-218-5p and FOXP1 in osteosarcoma cells.

### Inhibition of lncRNA HOXA-AS3 reduces migratory ability via miR-218-5p and FOXP1

**Figure 5:**
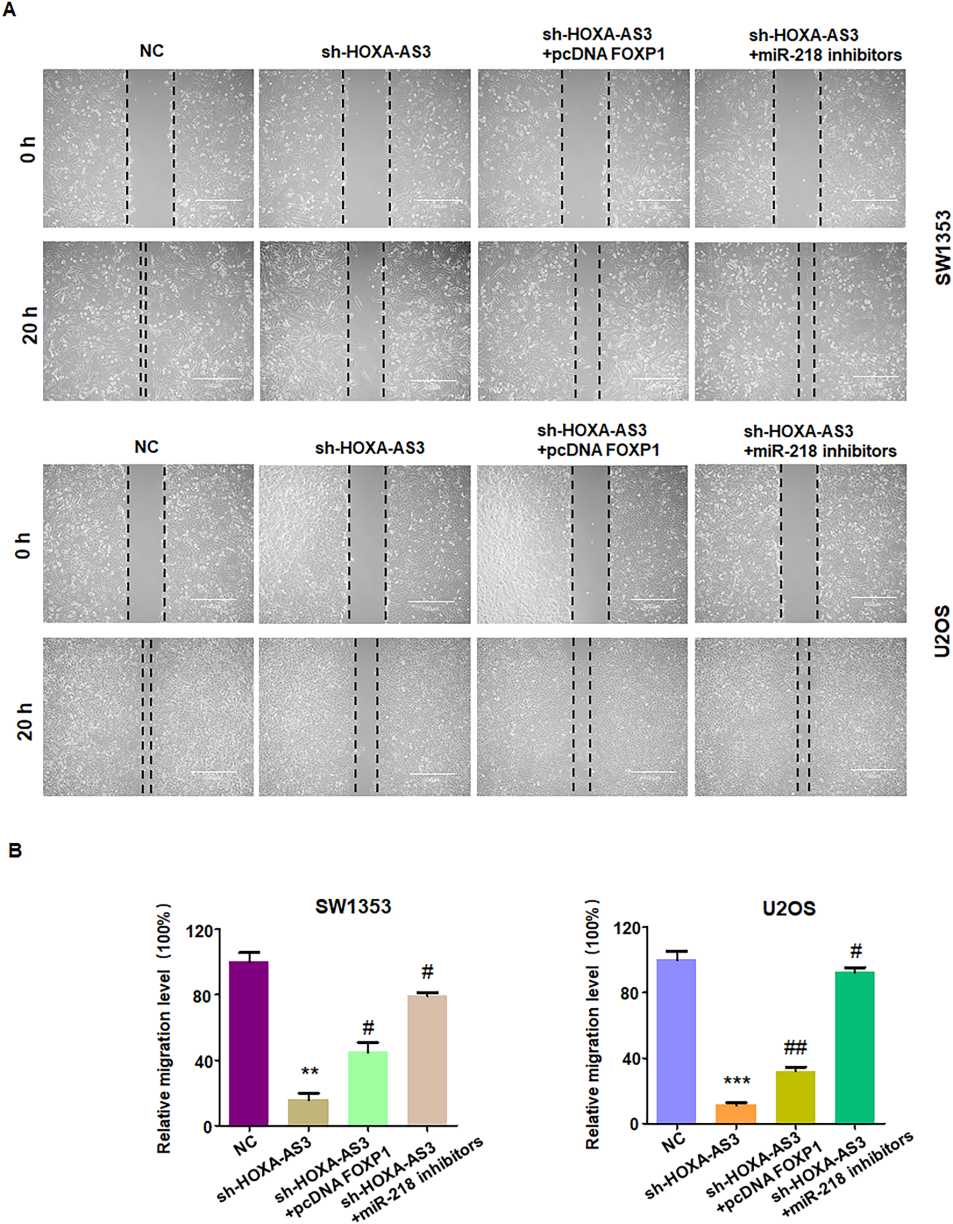
Downregulation of lncRNA HOXA-AS3 reduces cell migration via miR-218-5p and FOXP1. (**A**) Wound healing assays were performed to measure migratory ability in osteosarcoma cells co-transfected with sh-HOXA-AS3 and pcDNA FOXP1, or sh-HOXA-AS3 and miR-218 inhibitors. (**B**) Quantitative data are shown for colony formation. **p < 0.01; ***p < 0.001 versus control group. ^#^*p* < 0.05; ^##^*p* < 0.01 versus sh-HOXA-AS3 alone.

Wound healing assay was performed to measure whether inhibition of lncRNA HOXA-AS3 regulates cell migration via regulation of miR-218-5p and FOXP1 in osteosarcoma cells. To address this question, co-transfection of sh-HOXA-AS3, miR-218-5p inhibitor, or pcDNA FOXP1 was done in osteosarcoma cells. We observed that inhibition of lncRNA HOXA-AS3 reduced would healing in SW1353 and U2OS cells (Fig. 5A,B). Moreover, miR-218-5p inhibitor transfection abrogated sh-HOXA-AS3-induced suppression of wound healing in osteosarcoma cells (Fig. 5A,B). Furthermore, pcDNA FOXP1 transfection abolished sh-HOXA-AS3-involved suppression of wound healing in osteosarcoma cells (Fig. 5A,B). Altogether, downregulation of lncRNA HOXA-AS3 reduced wound healing via miR-218-5p and FOXP1 in osteosarcoma cells.

### Suppression of lncRNA HOXA-AS3 inhibits invasive ability via miR-218-5p and FOXP1

**Figure 6:**
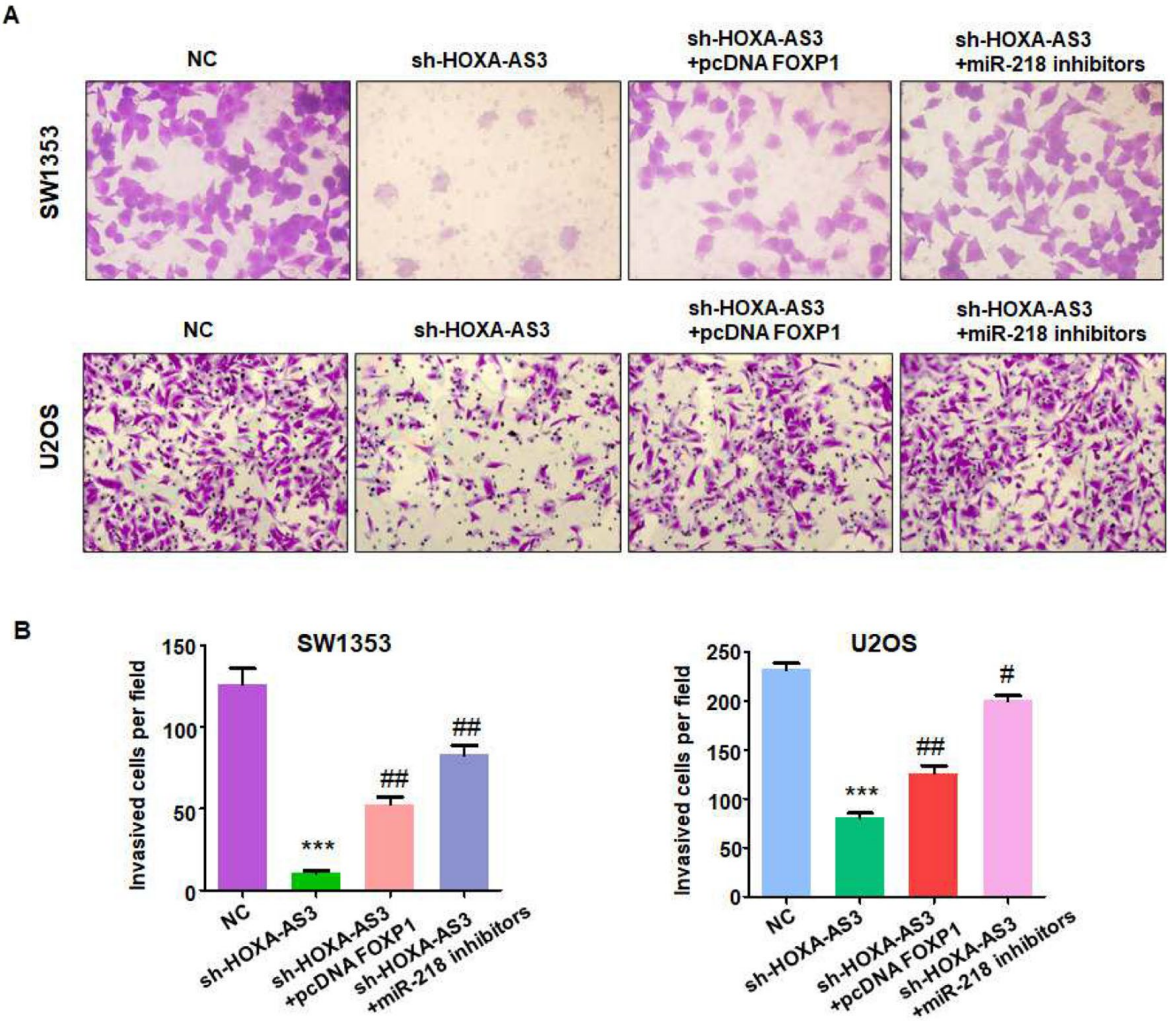
Downregulation of lncRNA HOXA-AS3 reduces cell invasion via miR-218-5p and FOXP1. (**A**) Transwell assays were performed to measure cell invasive ability in osteosarcoma cells co-transfected with sh-HOXA-AS3 and pcDNA FOXP1, or sh-HOXA-AS3 and miR-218 inhibitors. (**B**) Quantitative data are shown for colony formation. ***p < 0.001 versus control group. ^#^*p* < 0.05; ^##^*p* < 0.01 versus sh-HOXA-AS3 alone.

To test whether lncRNA HOXA-AS3 inhibition attenuated invasive ability via regulation of miR-218-5p and FOXP1, osteosarcoma cells were co-transfected with sh-HOXA-AS3 and miR-218-5p inhibitor, or sh-HOXA-AS3 and pcDNA FOXP1. The results from the Transwell assay showed that sh-HOXA-AS3 transfection reduced cell invasive ability in osteosarcoma cells (Fig. 6A,B). This phenotype was rescued by downregulation of miR-218-5p by its inhibitors (Fig. 6A,B). Similarly, overexpression of FOXP1 also rescued sh-HOXA-AS3-mediated suppression of cell invasive ability in osteosarcoma cells. Hence, silencing of lncRNA HOXA-AS3 reduced cell invasive ability in part via miR-218-5p and FOXP1 in osteosarcoma cells.

## Discussion

Evidence has revealed that lncRNAs are critical in regulating oncogenesis and drug resistance in human cancer^38–41^. LncRNA SATB2-AS1 targeted the expression of SATB2 and increased the tumor metastasis and proliferation via influencing the tumor microenvironment in osteosarcoma^42^. LncRNA XLOC_006786 inhibited tumor invasion, metastasis and proliferation via targeting miR-491-5p and Notch3 pathway in osteosarcoma^43^. LncRNA HOTAIRM1 regulated miR-664b-3p/Rheb/mTOR pathway and caused induction of proliferation via aerobic glycolysis in osteosarcoma^44^. LncRNA SNHG14 repressed ferroptosis and caused nutlin3a resistance through regulation of the miR-206 and SLC7A11 in osteosarcoma^45^. LncRNA EBLN3P regulated the expression of O-GlcNAc pathway via sponging miR-200a-3p, leading to methotrexate resistance in osteosarcoma cells^46^. Our previous study showed that lncRNA SCAMP1 regulated miR-26a-5p and ZEB2, contributing to promotion of cell invasion and viability in osteosarcoma^47^. These findings suggested that lncRNAs are involved in osteosarcoma development and progression.

LncRNA HOXA-AS3 was identified in pediatric gliomas as a biomarker for predicting prognosis^48^. LncRNA HOXA-AS3 regulated the EMT pathway and triggered tumor progression in epithelial ovarian cancer^49^. Moreover, lncRNA HOXA-AS3 regulated the miR-29a-3p and modulated tumor progression in cervical cancer. LncRNA HOXA-AS3 expression displayed a prognostic value in cervical cancer patients^32^. Furthermore, lncRNA HOXA-AS3 increased cell proliferation via modulation of miR-218-5p expression in oral squamous cell carcinoma^50^. Additionally, lncRNA HOXA-AS3 targeted the miR-4319 and SPNS2 expression, which caused the malignant progression in colorectal cancer^51^. Downregulation of lncRNA HOXA-AS3 blocked the tumor development via sponging miR-29c and upregulating CDK6 in pancreatic cancer^52^. One group reported that lncRNA HOXA-AS3 facilitated tumor progression via activation of NF-κB pathway and targeting the miR-29a-3p and LTβR in gastric cancer^33^. Another group reported that lncRNA HOXA-AS3 regulated the miR-455-5p and USP3 expression and enhanced malignant progression in glioblastoma multiforme^53^. In line with these findings, our study reported that lncRNA HOXA-AS3 facilitated cell proliferation and invasion via targeting miR-218-5p in osteosarcoma.

Several studies revealed that miR-218-5p inhibited cell proliferation, migration and invasion in MG63, U2OS and 143B osteosarcoma cells^35,36^. One study showed that miR-218-5p was involved in doxorubicin resistance in HOS and U2OS osteosarcoma cells^34^. In the current study, we identified the tumor suppressive function of miR-218-5p in U2OS and SW1353 osteosarcoma cells. LncRNA HOXA-AS3 has been reported to regulate the chemotherapeutic drug resistance in human cancer. For example, RNA sequencing analysis identified the numerous lncRNAs and miRNAs in small cell lung cancer, which are related with chemotherapy insensitivity, including lncRNA HOXA-AS3^54^. In non-small-cell lung carcinoma (NSCLC), lncRNA HOXA-AS3 interacted with HOXA3 and regulated EMT (epithelial-mesenchymal transition) and led to cisplatin resistance^55^. In bladder cancer, lncRNA HOXA-AS3 reduced chemosensitivity via working as a ceRNA to sponge miR-455-5p and increase the expression of Notch1^56^. One study revealed that depletion of lncRNA HOXA-AS3 reduced cell proliferation, migration, invasion in osteosarcoma cells via targeting miR-1286/TEAD1 axis. Overexpression of HOXA-AS3 was found in osteosarcoma tissues and was linked to poor outcomes^31^. Consistently, our study supported the oncogenic role of HOXA-AS3 in osteosarcoma cells. Furthermore, our study demonstrated that lncRNA HOXA-AS3 enhanced tumor progression via targeting miR-218-5p/FOXP1 in osteosarcoma.

In conclusion, lncRNA HOXA-AS3 regulated the expression of FOXP1 via sponging miR-218-5p, which led to promotion of colony formation, migration and invasion in osteosarcoma. Several limitations should be mentioned in this study. The mouse model is required to determine whether lncRNA HOXA-AS3 facilitates tumor growth in osteosarcoma. It is necessary to measure the expression of lncRNA HOXA-AS3 in osteosarcoma tissue samples and determine the association between HOXA-AS3 expression and prognosis in osteosarcoma patients. It is unclear whether lncRNA HOXA-AS3 could promote drug resistance in osteosarcoma, which is required to fully explore in the future. Taken together, targeting lncRNA HOXA-AS3 could be an alternative strategy to treat osteosarcoma. It is important to note that using suppression of lncRNA HOXA-AS3 expression is still premature for treating osteosarcoma patients. This could be due to targeting issues, delivery issues, safety and side effects. LncRNA HOXA-AS3 could regulate the expression of numerous downstream targets. The effective delivery of lncRNAs or their inhibitors to target cells remains a technical challenge. One lncRNA affects multiple signaling pathways and governs several cellular functions. Regulation of lncRNAs could affect normal cell function and have potential genotoxicity. Hence, there are relatively few clinical trials and studies on the role of lncRNAs in human cancer treatment.

## Materials and methods

### Cell culture

The U2OS human osteosarcoma cell line and SW1353 chondrosarcoma cell line were obtained from the cell bank of the Shanghai Cell Institute. The U2OS and SW1353 cells were cultured in Dulbecco’s Modified Eagle Medium (DMEM) with 1% penicillin–streptomycin and 10% fetal bovine serum. All cells were maintained at 37 °C incubator with 5% CO_2_.

### Transfection

The osteosarcoma U2OS and SW1353 cells were cultured in 6-well plates. Osteosarcoma cells were transfected with sh-HOXA-AS, shNC, 100 nM miR-218 inhibitor, 50 nM miR-218 mimics (Gene-Pharma CO. Ltd, Shanghai, China) by Lipofectamine 2000 as described previously^57^. Then, the transfected cells were further analyzed for their viability, invasion and migration.

### Quantitative real-time reverse transcription-PCR

TRLzol reagent was used to extract total RNA from the transfected osteosarcoma cells. 1 µg of RNA was used to obtain cDNA via reverse transcription. PCR was performed by SYBR Green Kit as described before^47^. The expression of GAPDH was for normalization. The primer sequences were as follows: HOXA-AS3: Forward CAC CTC TCT CAT CGA AAA AA CG, Reverse GCA CCA GGA AAG AGG ACA ATTC; miR-218-5p: Forward AGC GAG ATT TTC TGT TGT GCT T, Reverse GAC GTT CCA TGG TGC TTG AC; GAPDH, Forward TGT GGG CAT CAA TGG ATT TGG; Reverse ACA CCA TGT ATT CCG GGT CAA T. The thermocycling conditions of qPCR were as follows: for HOXA-AS3, 5 min at 95 °C, followed by 40 cycles of 95 °C for 30 s and 65 °C for 45 s; for miR-218-5p, 5 min at 95 °C, followed by 40 cycles at 95 °C for 15 s, 60 °C for 30 s, and 72 °C for 30 s.

### Colony formation assay

The transfected U2OS and SW1353 osteosarcoma cells were cultured in 6-well plates and maintained for 14 days at 37 °C incubator with 5% CO_2_. After the cells were washed by PBS four times, 4% paraformaldehyde was added to fix for 45 min. 0.1% crystal violet was used to stain the colonies for 20 min. Microscope was used to take images and the colony numbers were counted.

### Transwell invasion assay

The transfected U2OS and SW1353 osteosarcoma cells (5 × 10^4^ cells) were cultured in top level of Transwell plates. The top level of plates was filled with serum-free DMEM, while the bottom level of Transwell plates was added DMEM with 10% FBS. After 20 h, we removed the culture medium and cells on the top level of plates. Then, the bottom plates were stained using 0.1% crystal violet after the invaded cells were fixed by 4% paraformaldehyde for 30 min. An inverted microscope was used to take images for the invaded cells.

### Wound healing assay

The transfected U2OS and SW1353 osteosarcoma cells were cultured on 6-well plates. The 200 µl pipette tip was used to produce a wound after cells reached about 95% fluence. PBS was used to remove the floatage cells. Microscope was used to take images near the wound area after 20 h as described before^58^.

### Western blotting analysis

The transfected U2OS and SW1353 osteosarcoma cells were lysed using RIPA lysis buffer to extract total protein. We denatured the protein by boiling for 5 min. Then, 30 µg proteins were subjected to 10% SDS-PAGE gels at 100 voltage for 90 min, and then transferred to PVDF membrane at 45 voltage overnight. 5% non-fat dry milk was used to block the membrane. The primary antibody FOXP1 (1:2000, #2005, Cell Signaling Technology) were used to incubate the membrane at cold room overnight. GAPDH (1:5000, #2118, Cell Signaling Technology) was the loading control. Western blotting analysis were performed as described previously (Supplementary information)^59^.

### Luciferase report assay

Luciferase report assay was performed to determine the interaction between lncRNAs and miRNAs in this study. The mutant binding sequences of miR-218 in lncRNA HOXA-AS3 were cloned into pmirGLO dual-luciferase vector. The plasmids were transfected into U2OS and SW1353 cells. The luciferase activity was determined as described before^47^.

### Statistical analysis

All results were analyzed by GraphPad Prism 5.0. Student t-test was used to determine the significant difference between two groups. Analysis of variance (ANOVA) was used to determine the significant difference between three or more groups. Means ± SD was used to represent the results. *P* < 0.05 was considered as statistically significant.

### Supplementary Information

Supplementary Figure 1.

## Data Availability

Data are available upon reasonable request. To request access to the data, please send an email to dl_xjmu@126.com.

## References

[1] Panez-Toro I (2023). Advances in osteosarcoma. Curr. Osteoporos. Rep..

[2] Bryan JN (2024). Updates in osteosarcoma. Vet. Clin. North Am. Small Anim. Pract..

[3] Beird HC (2022). Osteosarcoma. Nat. Rev. Dis. Primers.

[4] Basit Q, Qazi HS, Tanveer S (2023). Osteosarcoma and its advancement. Cancer Treat. Res..

[5] Gill J, Gorlick R (2021). Advancing therapy for osteosarcoma. Nat. Rev. Clin. Oncol..

[6] Li S, Zhang H, Liu J, Shang G (2023). Targeted therapy for osteosarcoma: A review. J. Cancer Res. Clin. Oncol..

[7] Cheng S, Wang H, Kang X, Zhang H (2024). Immunotherapy innovations in the fight against osteosarcoma: Emerging strategies and promising progress. Pharmaceutics.

[8] Kansara M, Teng MW, Smyth MJ, Thomas DM (2014). Translational biology of osteosarcoma. Nat. Rev. Cancer.

[9] Gianferante DM, Mirabello L, Savage SA (2017). Germline and somatic genetics of osteosarcoma—Connecting aetiology, biology and therapy. Nat. Rev. Endocrinol..

[10] Twenhafel L, Moreno D, Punt T, Kinney M, Ryznar R (2023). Epigenetic changes associated with osteosarcoma: A comprehensive review. Cells.

[11] Yao Y, Wang D, Zheng L, Zhao J, Tan M (2024). Advances in prognostic models for osteosarcoma risk. Heliyon.

[12] Kristensen LS, Jakobsen T, Hager H, Kjems J (2022). The emerging roles of circRNAs in cancer and oncology. Nat. Rev. Clin. Oncol..

[13] Ferrer J, Dimitrova N (2024). Transcription regulation by long non-coding RNAs: Mechanisms and disease relevance. Nat. Rev. Mol. Cell Biol..

[14] Zhang MW (2021). Comprehensive circRNA-microRNA-mRNA network analysis revealed the novel regulatory mechanism of *Trichosporon asahii* infection. Mil. Med. Res..

[15] Winkle M, El-Daly SM, Fabbri M, Calin GA (2021). Noncoding RNA therapeutics—Challenges and potential solutions. Nat. Rev. Drug. Discov..

[16] Liu SJ, Dang HX, Lim DA, Feng FY, Maher CA (2021). Long noncoding RNAs in cancer metastasis. Nat. Rev. Cancer.

[17] Zeng L, Liu L, Ni WJ, Xie F, Leng XM (2023). Circular RNAs in osteosarcoma: an update of recent studies. Int. J. Oncol..

[18] Hussen BM (2023). Targeting miRNA by CRISPR/Cas in cancer: advantages and challenges. Mil. Med. Res..

[19] Yao Q (2022). The integrated comprehension of lncRNA HOXA-AS3 implication on human diseases. Clin. Transl. Oncol..

[20] Chong ZX, Ho WY, Yeap SK (2024). Tumour-regulatory role of long non-coding RNA HOXA-AS3. Prog. Biophys. Mol. Biol..

[21] Zhu XX (2016). Long non-coding RNA HoxA-AS3 interacts with EZH2 to regulate lineage commitment of mesenchymal stem cells. Oncotarget.

[22] Zhu X (2019). Long Noncoding RNA HOXA-AS3 integrates NF-kappaB signaling to regulate endothelium inflammation. Mol. Cell Biol..

[23] Gu C (2021). Exploring the potential key IncRNAs with endometriosis by construction of a ceRNA network. Int. J. Gen. Med..

[24] Li ZK, Gao LF, Zhu XA, Xiang DK (2021). LncRNA HOXA-AS3 promotes the progression of pulmonary arterial hypertension through mediation of miR-675-3p/PDE5A axis. Biochem. Genet..

[25] Chi K (2020). Knockdown of lncRNA HOXA-AS3 suppresses the progression of atherosclerosis via sponging miR-455-5p. Drug Des. Dev. Ther..

[26] Wu F (2017). Upregulation of long noncoding RNA HOXA-AS3 promotes tumor progression and predicts poor prognosis in glioma. Oncotarget.

[27] Zhang H (2018). Increased levels of the long noncoding RNA, HOXA-AS3, promote proliferation of A549 cells. Cell Death Dis..

[28] Tong Y (2019). LncRNA HOXA-AS3 sponges miR-29c to facilitate cell proliferation, metastasis, and EMT process and activate the MEK/ERK signaling pathway in hepatocellular carcinoma. Hum. Gene Ther. Clin. Dev..

[29] Zeng C (2021). HOXA-AS3 promotes proliferation and migration of hepatocellular carcinoma cells via the miR-455-5p/PD-L1 axis. J. Immunol. Res..

[30] Peng S (2018). An overview of long noncoding RNAs involved in bone regeneration from mesenchymal stem cells. Stem Cells Int..

[31] Xiao X (2023). Long non-coding HOXA-AS3 contributes to osteosarcoma progression through the miR-1286/TEAD1 axis. J. Orthop. Surg. Res..

[32] Xu H, Tang Y, He C, Tian Y, Ni R (2022). Prognostic value of lncRNA HOXA-AS3 in cervical cancer by targeting miR-29a-3p and its regulatory effect on tumor progression. J. Obstet. Gynaecol. Res..

[33] Qu F, Zhu B, Hu YL, Mao QS, Feng Y (2021). LncRNA HOXA-AS3 promotes gastric cancer progression by regulating miR-29a-3p/LTbetaR and activating NF-kappaB signaling. Cancer Cell Int..

[34] Wei W, Ji L, Duan W, Zhu J (2020). CircSAMD4A contributes to cell doxorubicin resistance in osteosarcoma by regulating the miR-218-5p/KLF8 axis. Open Life Sci..

[35] Pan F (2020). The novel circ_0028171/miR-218-5p/IKBKB axis promotes osteosarcoma cancer progression. Cancer Cell Int..

[36] Jia G, Wang Y, Yu Y, Li Z, Wang X (2020). Long non-coding RNA NR2F1-AS1 facilitates the osteosarcoma cell malignant phenotype via the miR-485-5p/miR-218-5p/BIRC5 axis. Oncol. Rep..

[37] Li H (2021). FOXP1 drives osteosarcoma development by repressing P21 and RB transcription downstream of P53. Oncogene.

[38] Song Z (2022). LncRNA MALAT1 regulates METTL3-mediated PD-L1 expression and immune infiltrates in pancreatic cancer. Front. Oncol..

[39] Liu J, Shang G (2022). The roles of noncoding RNAs in the development of osteosarcoma stem cells and potential therapeutic targets. Front. Cell. Dev. Biol..

[40] Jiang W, Pan S, Chen X, Wang ZW, Zhu X (2021). The role of lncRNAs and circRNAs in the PD-1/PD-L1 pathway in cancer immunotherapy. Mol. Cancer.

[41] Xie W (2022). Emerging roles of long noncoding RNAs in chemoresistance of pancreatic cancer. Semin. Cancer Biol..

[42] Wang P (2023). LncRNA SATB2-AS1 promotes tumor growth and metastasis and affects the tumor immune microenvironment in osteosarcoma by regulating SATB2. J. Bone Oncol..

[43] Xu JY (2023). Long noncoding RNA XLOC_006786 inhibits the proliferation, invasion and metastasis of osteosarcoma cells through NOTCH3 signaling pathway by targeting miR-491-5p. Hum. Cell.

[44] Yu X (2023). LncRNA-HOTAIRM1 promotes aerobic glycolysis and proliferation in osteosarcoma via the miR-664b-3p/Rheb/mTOR pathway. Cancer Sci..

[45] Li L (2023). LncSNHG14 promotes nutlin3a resistance by inhibiting ferroptosis via the miR-206 /SLC7A11 axis in osteosarcoma cells. Cancer Gene Ther..

[46] Sun MX, An HY, Sun YB, Sun YB, Bai B (2022). LncRNA EBLN3P attributes methotrexate resistance in osteosarcoma cells through miR-200a-3p/O-GlcNAc transferase pathway. J. Orthop. Surg. Res..

[47] Li R (2022). LncRNA SCAMP1 disrupts the balance between miR-26a-5p and ZEB2 to promote osteosarcoma cell viability and invasion. Front. Oncol..

[48] Zhang J (2023). Identification and validation of a novel HOX-related classifier signature for predicting prognosis and immune microenvironment in pediatric gliomas. Front. Cell Dev. Biol..

[49] Eoh KJ (2023). HOXA-AS3 induces tumor progression through the epithelial-mesenchymal transition pathway in epithelial ovarian cancer. Oncol. Rep..

[50] Zhao Y, Yao R (2021). Long non-coding RNA HOXA-AS3 promotes cell proliferation of oral squamous cell carcinoma through sponging microRNA miR-218-5p. Bioengineered.

[51] Jiang Y, Yu XY, Sun HX, Gu XY, Geng JS (2021). Long non-coding RNA HOXA-AS3 facilitates the malignancy in colorectal cancer by miR-4319/SPNS2 axis. J. Physiol. Biochem..

[52] Zhang X, Zhu H, Qu X, Yu Z, Zhang J (2021). Suppressing LncRNA HOXA-AS3 by CRISPR-dCas9 inhibits pancreatic cancer development. J. Cancer.

[53] Chen W (2020). LncRNA HOXA-AS3 promotes the malignancy of glioblastoma through regulating miR-455-5p/USP3 axis. J. Cell Mol. Med..

[54] Kuang P (2020). RNA sequencing analysis of small cell lung cancer reveals candidate chemotherapy insensitivity long noncoding RNAs and microRNAs. Ann. Transl. Med..

[55] Lin S (2019). LncRNA HOXA-AS3 confers cisplatin resistance by interacting with HOXA3 in non-small-cell lung carcinoma cells. Oncogenesis.

[56] Chen D (2020). Reduction of bladder cancer chemosensitivity induced by the effect of HOXA-AS3 as a ceRNA for miR-455-5p that upregulates Notch1. Front. Oncol..

[57] Ding L (2021). MiR-506 exerts antineoplastic effects on osteosarcoma cells via inhibition of the Skp2 oncoprotein. Aging (Albany NY).

[58] Li R (2019). CBX7 inhibits cell growth and motility and induces apoptosis in cervical cancer cells. Mol. Ther. Oncol..

[59] Ding L (2018). S-phase kinase-associated protein 2 is involved in epithelial-mesenchymal transition in methotrexate-resistant osteosarcoma cells. Int. J. Oncol..

