# Supplementary figures and images for: LncRNA HOXA-AS3 promotes cell proliferation and invasion via targeting miR-218-5p/FOXP1 axis in osteosarcoma
Source: Sci Rep. 2024 Jul 17;14:16581. doi: 10.1038/s41598-024-67596-4 (PMC11254915; doi:10.1038/s41598-024-67596-4)

Fig 2E

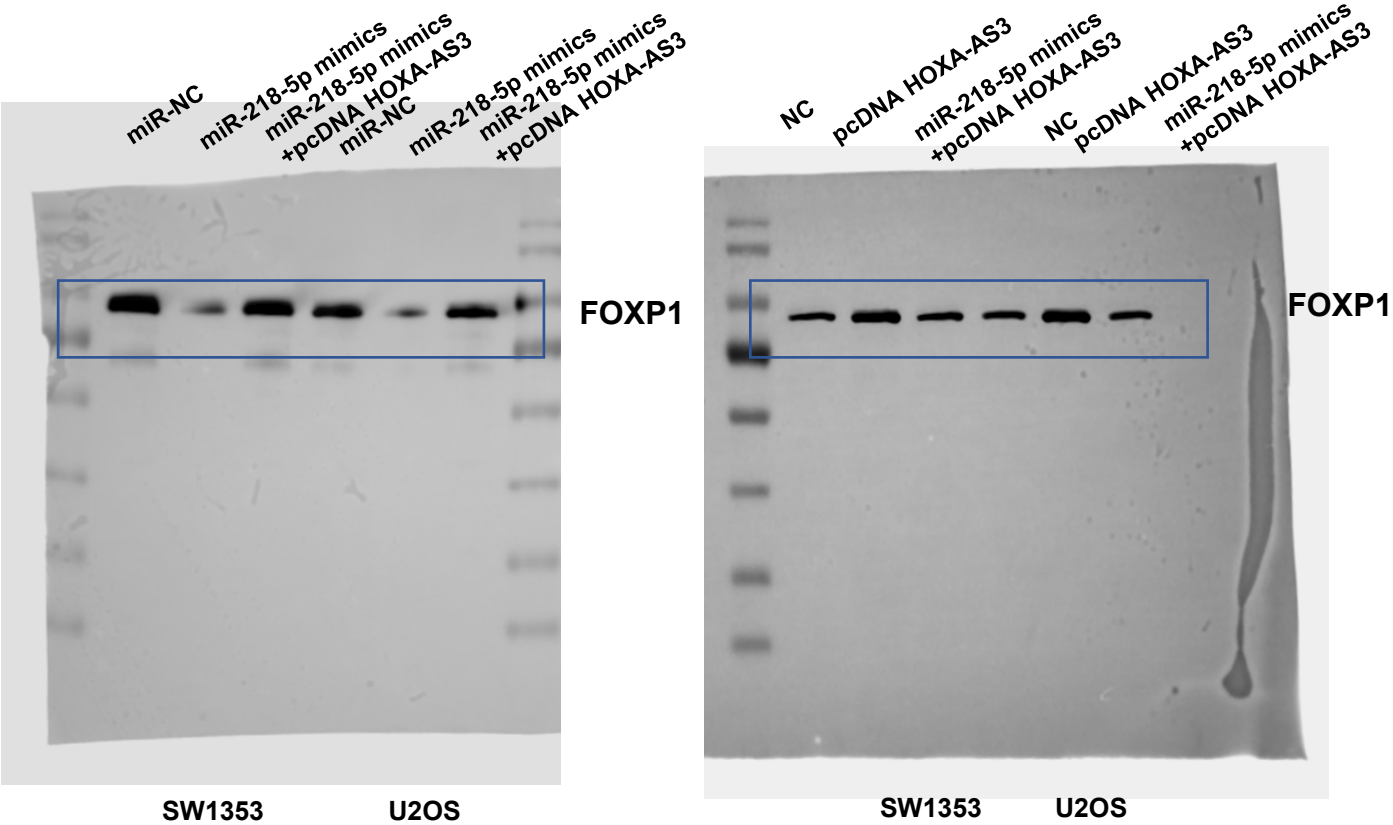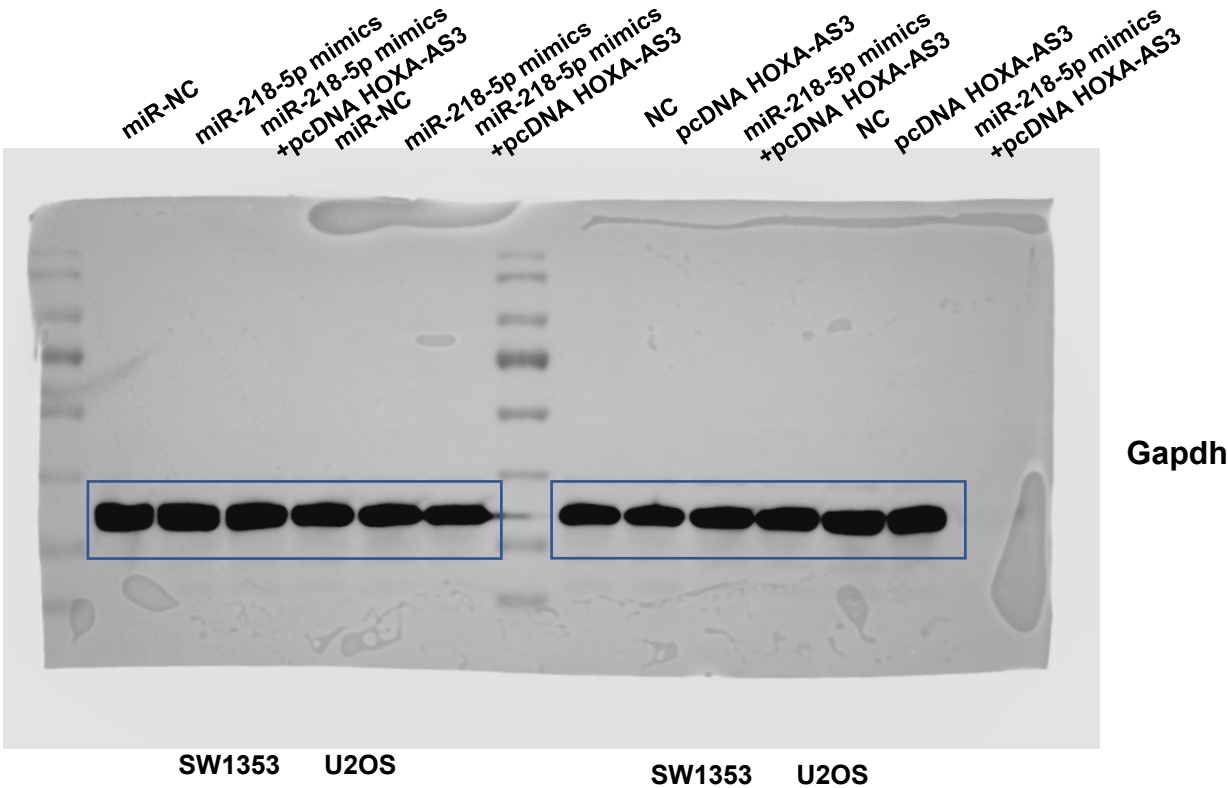

Supplement: Supplementary file 1 — Supplementary Figure 1. [file 41598_2024_67596_MOESM1_ESM.pdf]
